# The Expression of Methyltransferase-Like 3 in Oral Precancerous Lesions and Oral Squamous Cell Carcinoma

**DOI:** 10.1055/s-0042-1747950

**Published:** 2022-07-04

**Authors:** Chatchaphan Udompatanakorn, Patrayu Taebunpakul

**Affiliations:** 1 Department of Oral Surgery and Oral Medicine, Faculty of Dentistry, Srinakharinwirot University, Bangkok, Thailand

**Keywords:** oral epithelial dysplasia, oral squamous cell carcinoma, methyltransferase-like 3, immunohistochemistry

## Abstract

**Objective** N6-methyladenosine is the most frequent mRNA modification in eukaryotic cells. It is catalyzed by the methyltransferase complex, methyltransferase-like 3 (METTL3). Previous studies have revealed that METTL3 plays a role in various cancers. However, there is limited information about the roles of METTL3 in oral epithelial dysplasia (OED). This study determined METTL3 expression in normal oral mucosa (NOM), OED, and oral squamous cell carcinoma (OSCC) by immunohistochemistry.

**Materials and Methods** Twenty formalin-fixed paraffin embedded specimens each of NOM, OED, and OSCC were included. The expression pattern, the number of positive cells, the staining intensity, and the histochemical score (H-score) of METTL3 were investigated.

**Statistical Analysis** The data were analyzed by using one-way analysis of variance, chi-squared test, and a Kruskal–Wallis test. A *p-* value < 0.05 indicated statistically significant.

**Results** The METTL3 expression in NOM was observed in the basal, parabasal, and lower layers of epithelium. In low-grade OED, METTL3 was expressed in the lower epithelial layers and partially presented in the spinous layer. However, in high-grade OED, METTL3 expression was observed in the lower layers, spinous layers, and upper layers of dysplastic epithelium. For OSCC, METTL3 immunostaining was presented in both the peripheral and central cells of the tumor islands. All NOM samples showed weak-to-moderate METTL3 staining intensity, while the moderate-to-strong METTL3 staining intensity was observed in 95% of both OED and OSCC specimens ( *p* < 0.05). The percentage of METTL3 positive cells and H-score was highest in OSCC, followed by OED and NOM, respectively ( *p* < 0.05). Interestingly, H-score was greater in high-grade OED (209.8 ± 18.61) when compared with low-grade OED (162.1 ± 38.93) ( *p* < 0.05).

**Conclusion** METTL3 expression in OED and OSCC was more outstanding than in NOM, suggesting possible roles for OED and OSCC pathogenesis. Additionally, METTL3 expression may be an indicator for OED progression to OSCC.

## Introduction

Head and neck cancers are the seventh most common type of cancer in the world. 1 They arise from various locations such as the oral cavity, oropharynx, hypopharynx, nasopharynx, and larynx. 1 More than 90% of the oral cancers are oral squamous cell carcinomas (OSCCs). 2 OSCCs are usually preceded by leukoplakia, which is one of the common potentially malignant disorders of the oral cavity. 3 Previous study also reported that most of oral potentially malignant disorders, particularly erythroplakia and leukoplakia at tongue, showed evidence of epithelial dysplasia. 4 The presence of epithelial dysplasia in leukoplakia is an important prognostic factor for malignant transformation. 3 Despite the progress in cancer diagnosis and surgical treatment, the 5-year survival rate of OSCCs remains unfavorable (∼50%). 5 Thus, identifying reliable biomarkers to classify oral dysplastic lesions with a high risk of malignant transformation would establish opportunities for early therapeutic interventions. 5

Epigenetic alterations, such as DNA and RNA methylation, histone protein modification, and microRNA dysregulation, have been reported to contribute to the tumorigenesis of OSCCs. 6 N6-methyladenosine (m6A), methylated at the N6 position of adenosine, is one of the most common and abundant RNA modifications in humans. 7 This RNA modification is dynamic and reversible 7 and m6A can be methylated by methyltransferases (writers), removed by demethylases (erasers), and recognized by reader proteins. 7 The m6A modification is involved in various biological processes, such as embryonic development, spermatogenesis, and circadian clock controlling. 8 Abnormal m6A modification was found to be associated with human diseases and cancers. 7 8

Methyltransferase-like 3 (METTL3) was identified as a main methyltransferase of the methyltransferase complex, together with its associated proteins methyltransferase-like 14 (METTL14) and Wilms tumor 1 associated protein. 7 9 METTL3 plays a role in mRNA metabolism including splicing, decay, and the export of mRNA through m6A modification. 8 Several studies have reported that overexpression of METTL3 may promote tumor cell proliferation, migration, and the invasion of various solid tumors *in vitro* , such as gastric adenocarcinoma, and colorectal cancer. 10 11 In contrast, there were studies that showed the upregulation of METTL3, which could inhibit proliferation, migration, and invasion functions in colorectal cancer and renal cell carcinoma cells. 12 13 To date, a few studies have reported the expressions and possible roles of METTL3 in OSCCs. 14 15 16 17 18 These studies indicated that METTL3 was overexpressed in OSCC tissues or cell lines compared with normal mucosa tissues or normal oral keratinocyte cell lines. 14 15 16 17 18 Additionally, a higher METTL3 immunopositivity was significantly correlated with tumor size, advanced tumor stage, and lymph node metastasis in OSCC cases. 14 15 16 17 These studies suggested that METTL3 could promote the proliferation and migration of OSCC cells *in vitro* and tumor growth *in vivo* . 14 15 16 17 However, there are limited data on the role of METTL3 in oral precancerous lesions, especially in oral epithelial dysplasia (OED). Therefore, this study aimed to evaluate the expression of METTL3 in normal oral mucosa (NOM), OED (low- and high-grade OED) and OSCC using immunohistochemistry. The results from this study could contribute to a better understanding of the role of METTL3 in the malignant transformation process of OED.

## Materials and Methods

### Specimens

Sixty paraffin-embedded tissues, including 20 cases of OSCC (16 cases of well differentiated OSCC and 4 cases of moderately differentiated OSCC), 20 cases of OED (10 cases of high-grade OED and 10 cases of low-grade OED), and 20 cases of NOM (15 cases of healthy gingival tissue and 5 cases of normal epithelium overlying fibroma of buccal mucosa) were obtained from the Department of Oral Surgery and Oral Medicine, in the Faculty of Dentistry at Srinakharinwirot University from 2010 to 2019. The OED specimens were graded based on the criteria of the World Health Organization 2017. 19 The healthy gingival tissues were obtained during the third molar surgical removal process from volunteers, who signed written informed consent. All of the selected specimens were histologically diagnosed by oral pathologists. The clinical data of all specimens, including age, gender, lesion site, and T-stage (for OSCC) were also collected from the records. The research project was approved by the Ethical Committee for Research in Human Subjects of Srinakharinwirot University, no. SWUEC/X-463/2563.

### Immunohistochemistry

Briefly, paraffin-embedded specimens (4 μm) were deparaffinized and rehydrated. The immunohistochemistry was performed using EnVision kit (Dako Agilent, California, United States). Antigen retrieval was performed in Tris-ethylenediaminetetraacetic acid buffer pH 9.0 by using a microwave (700 Watts) for 10 minutes. After 20 minutes of cooling down to room temperature, and endogenous peroxidase activity was blocked for 30 minutes of incubation. The sections were thoroughly washed with wash buffer (Dako Agilent, California, United States). The sections were incubated overnight at 4°C with the primary antibody against METTL3 (ab195352, abcam, Cambridge, United Kingdom) at a concentration of 1:250. The secondary antibody was added to the sections for 30 minutes. After washing with wash buffer, chromogen,3,3I-diaminobenzidine was applied for 5 minutes. Finally, the sections were dehydrated, and mounted using the Bio Mount HM mounting medium (Bio-Optica, Milan, Italy). For negative staining control, the protein block (5% bovine serum albumin in Tris-Buffered Saline with Tween 20) without primary antibodies was added to specimens. Human bladder cancer (catalog no. T2235010, Biochain Institute Inc., California, United States) and normal rat testis tissue were taken as positive control.

### Interpretation of Immunostaining

The expression of METTL3 was evaluated based on the expression pattern, the number of positive cells, and the staining intensity. Additionally, the histochemical scoring (H-score) was used for interpretation. 20 The photographs were taken from the epithelium tissues of each slide with a 100x magnification using Motic Microscope camera (Motic, Fujian, China). All the photographs were scored using ImageJ software (NIH, Maryland, United States). At least 1,000 cells were counted on each slide. The METTL3 expression showed the intensities of the color brown as the nuclear staining in the epithelium was counted as positive. The staining intensity score was graded as: 0 = no evidence of staining, 1 = weak staining, 2 = moderate staining, and 3 = strong staining. The H-score was determined by adding the results of the multiplication of the percentage of cells with staining intensity ordinal values with 300 possible values. 20 The specimens were analyzed and scored by an oral pathologist (CU). Additionally, the staining of METTL3 was categorized as high METTL3 expression (H-score > 150) and low METTL3 expression (H-score ≤ 150).

### Statistical Analysis

The statistical analysis was performed using GraphPad Prism Version 9 software for Windows (GraphPad Software, California, United States). The percentage of positive cells, the score of staining intensity, and the H-score among groups (NOM, OED, and OSCC) were compared using one-way analysis of variance, chi-squared test, and Kruskal–Wallis test, respectively. The comparison between two groups for the percentage of positive cells and H-scores assessed using Tukey's and Dunn's multiple comparison test, respectively. The statistically significant differences between the percentage of positive cells, the score of staining intensity, and the H-score between low- versus high-grade OED and differentiated OSCC versus moderately-to-poorly differentiated OSCC were calculated using an unpaired *t* -test, chi-squared test, or Mann–Whitney test. The correlations between METTL3 and the clinicopathological variables of the OED and OSCC samples were analyzed using Fisher's exact test. The statistical significance was defined as a *p* -value of <0.05.

## Results

### Demographic and Clinical Data of the Study Groups

The demographic and clinical data of the study groups were presented in Table 1 . The mean ages of OED and OSCC were 58.8 ± 10.75 and 61.7 ± 16.27, respectively. However, in the NOM group, the mean age was lower at 35.1 ± 13.24. The male to female ratio for NOM, OED, and OSCC was 3:2, 2:3, and 5.67:1, respectively. The differences for age and sex distribution among groups were noted ( *p* < 0.05).

**Table 1:** Demographic and clinical data of the study groups

Clinicopathological features	NOM *n* (%)	OED *n* (%)	OSCC *n* (%)	*p* -Value
Age (years old)				
Mean ± SD	35.1 ± 13.24	58.8 ± 10.75	61.7 ± 16.27	< 0.05
Range	21–64	41–82	28–83	
≤ 60	18 (90)	14 (70)	10 (50)	
> 60	2 (10)	6 (30)	10 (50)	
Gender				
Male	12 (60)	8 (40)	17 (85)	< 0.05
Female	8 (40)	12 (60)	3 (15)	
Total	20 (100)	20 (100)	20 (100)	
Site of lesions				
Gingiva	15 (75)	2 (10)	11 (55)	N/A
Buccal mucosa	5 (25)	4 (20)	2 (10)	
Tongue	0 (0)	10 (50)	5 (25)	
Hard palate	0 (0)	4 (20)	1 (5)	
Floor of the mouth	0 (0)	0 (0)	1 (5)	
Total	20 (100)	20 (100)	20 (100)	
T stage of OSCC				
T1	N/A	N/A	12 (60)	N/A
T2 and T3	N/A	N/A	8 (40)	
Total	N/A	N/A	20 (100)	
Histology of OED				
Low-grade OED	N/A	10 (50)	N/A	N/A
High-grade OED	N/A	10 (50)	N/A	
Total	N/A	20 (100)	N/A	
Histology of OSCC				
Well differentiated	N/A	N/A	16 (80)	N/A
Moderately-to-poorly differentiated	N/A	N/A	4 (20)	
Total	N/A	N/A	20 (100)	

Abbreviations: N/A, not available; NOM, normal oral mucosa; OED, oral epithelial dysplasia; OSCC, oral squamous cell carcinoma; SD, standard deviation.

*p*^a^ = NOM; ^b^ = OED; ^c^ = OSCC; ^a,b,c^ represent comparisons between two or more groups for statistical differences;  < 0.05 indicated statistically significant differences.

The most common site of OED and OSCC lesion was found in the tongue (50%), and gingiva (55%), respectively. The NOM specimens were obtained from gingiva (75%) and buccal mucosa (25%). In the OSCC group, 12 cases (60%) were categorized as T1, while 8 cases (40%) were categorized as T2 and T3. The OED group was graded histologically into 10 cases of low-grade OED (50%), and 10 cases of high-grade OED (50%). Additionally, the histological features of OSCC cases consisted of 16 cases (80%) of well-differentiated OSCC, and 4 cases (20%) of moderately-to-poorly differentiated OSCC.

### The Expression Pattern of METTL3

**Fig. 1:**
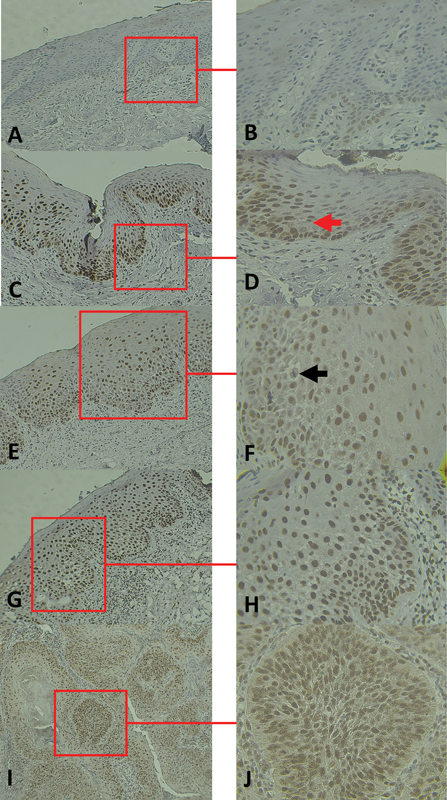
Methyltransferase-like 3 (METTL3) expression in the representative of the normal oral mucosa (NOM) ( **A, B** ), low-grade oral epithelial dysplasia (OED) ( **C, D** ), high-grade (moderate) OED ( **E** , **F** ) and high-grade OED (carcinoma *in situ* ) ( **G** , **H** ), and well-differentiated oral squamous cell carcinoma (OSCC) ( **I** , **J** ). The expression of METTL3 in NOM was found mainly in basal and parabasal layers of the squamous epithelium ( **A** , **B** ). In low-grade or mild epithelial dysplasia, METTL3 immunostaining was presented in the lower epithelial layers and partially presented in the spinous layer of the epithelium ( **C** , **D** ). Red arrow indicated mitosis in the lower layers of the squamous epithelium ( **D** ). The expression of METTL3 in high-grade OED was found in the lower layers, spinous layers, and upper part of dysplastic epithelium ( **E** – **H** ). Carcinoma *in situ* cases ( **G** , **H** ) showed higher number of the METTL3 positive cells compared with the moderate OED ( **E** , **F** ). Black arrow indicated mitosis in the middle layers of the squamous epithelium ( **F** ). In well-differentiated OSCC, nuclear expression of METTL3 was observed in the central and peripheral area of tumor islands. Keratin pearl was found to be negative for METTL3 immunoexpression ( **I** , **J** ). Original magnification × 100 ( **A** – **I** ), × 400 ( **B** – **J** ).

All specimens were expressed METTL3. Immunopositivity for METTL3 was observed only in the nucleus of samples. For NOM, the METTL3 immunostaining was found in the basal, parabasal, and lower layers of epithelium. The METTL3 positive cells in OED and OSCC were higher than NOM. In low-grade OED, METTL3 immunostaining was presented in the lower epithelial layers and partially presented in the spinous layer of the epithelium. Interestingly, in the high-grade OED, METTL3 positive cells were presented in the lower layers, spinous layers, and upper layers of dysplastic epithelium. For OSCC, METTL3 immunostaining was observed in both peripheral and central cells of the tumor islands ( Fig. 1 ).

### The Percentage of METTL3 Positive Cells, Staining Intensity, and H-Score

OSCC samples showed the highest mean percentage of positive cells (88.9% ± 6.39%), followed by OED (77.5% ± 8.40%), and NOM (24.1% ± 9.33%). There was a statistically significant difference in the mean percentage of METTL3 positive cells among these groups ( *p* < 0.001) ( Table 2 ). In addition, the mean percentage of METTL3 positive cells in high-grade OED (82.3% ± 7.37%) was significantly greater than low-grade OED (72.6% ± 6.50%) ( *p* < 0.05) ( Table 3 ). However, there were no statistically significant differences between the mean percentage of METTL3 positive cells between well differentiated OSCC and moderately-to-poorly differentiated OSCC ( *p* = 0.32) ( Table 3 ).

**Table 2:** The expression of METTL3 in NOM, OED, and OSCC

METTL3	NOM ^a^ *n* (%)	OED ^b^ *n* (%)	OSCC ^c^ *n* (%)	*p* -Value
No. of positive cases	20 (100)	20 (100)	20 (100)	N/A
No. of negative cases	0 (0)	0 (0)	0 (0)	N/A
Percentage of positive cells (mean ± SD)	24.1 ± 9.33	77.5 ± 8.40	88.9 ± 6.39	*p* < 0.001 ^abc, ab, ac^ *p* < 0.05 ^bc^
Intensity				
Score 0	0 (0)	0 (0)	0 (0)	*p* < 0.001 ^abc^
Score 1	10 (50)	1 (5)	1 (5)	
Score 2	10 (50)	10 (50)	3 (15)	
Score 3	0 (0)	9 (45)	16 (80)	
H-score (mean ± SD)	39.0 ± 24.49	186.0 ± 47.33	244.3 ± 52.85	*p* < 0.001 ^abc, ab, ac^ *p* = 0.06 ^bc^

Abbreviations: H-score, histochemical score; METTL3, methyltransferase-like 3; N/A, not available; NOM, normal oral mucosa; OED, oral epithelial dysplasia; OSCC, oral squamous cell carcinoma; SD, standard deviation.

^a^ = NOM; ^b^ = OED; ^c^ = OSCC; ^a,b,c^ represent comparisons between two or more groups for statistical differences; *p* < 0.05 indicated statistically significant differences.

**Table 3:** The expression of METTL3 in low- and high-grade OED and well-differentiated and moderately-to-poorly differentiated OSCC

METTL3	OED		*p* -Value	OSCC		*p* -Value
	Low-grade *n* (%)	High-grade *n* (%)		WD *n* (%)	MD to PD *n* (%)	
No. of positive cases	10 (100)	10 (100)	N/A	16 (100)	4 (100)	N/A
No. of negative cases	0 (0)	0 (0)	N/A	0 (0)	0 (0)	N/A
Percentage of positive cells (mean ± SD)	72.6 ± 6.50	82.3 ± 7.37	< 0.05	88.13 ± 6.87	91.75 ± 2.87	0.32
Intensity						
Score 0	0 (0)	0 (0)	0.30	0 (0)	0 (0)	0.74
Score 1	1 (10)	0 (0)		1 (6.25)	0 (0)	
Score 2	6 (60)	4 (40)		2 (12.5)	1 (25)	
Score 3	3 (30)	6 (60)		13 (81.25)	3 (75)	
H-score (mean ± SD)	162.1 ± 38.93	209.8 ± 18.61	< 0.05	242.2 ± 55.31	252.5 ± 47.46	0.60

Abbreviations: H-score, histochemical score; METTL3, methyltransferase-like 3; MD to PD, moderately-to-poorly differentiated; N/A, not available; OED, oral epithelial dysplasia; OSCC, oral squamous cell carcinoma; SD, standard deviation; WD, well-differentiated.

*p* < 0.05 indicated statistically significant differences.

For the staining intensity, all NOM samples showed weak-to-moderate METTL3 staining intensity, while moderate-to-strong METTL3 staining intensity was observed in 95% of both OED and OSCC specimens. The differences in METTL3 staining intensity were observed among groups ( *p* < 0.001) ( Table 2 ). Nevertheless, there was no statistically significant difference between the METTL3 staining intensity between low- and high-grade OED ( *p* = 0.30), and between well differentiated OSCC and moderately-to-poorly differentiated OSCC ( *p* = 0.74) ( Table 3 ).

The H-score of METTL3 expression in OED (186.0 ± 47.33) and OSCC (244.3 ± 52.85) was significantly greater than NOM (39.0 ± 24.49) ( *p* < 0.001) ( Table 2 ). However, there was no significant difference between the H-score of the OED and OSCC groups ( *p* = 0.06) ( Table 2 ). Interestingly, the METTL3 H-score was greater than the high-grade OED (209.8 ± 18.61) than low-grade OED (162.1 ± 38.93) ( *p* < 0.05) ( Table 3 ). However, no statistically significant difference was observed between the METTL3 H-score of well differentiated OSCC and moderately-to-poorly differentiated OSCC ( *p* = 0.60) ( Table 3 ).

### Correlations between METTL3 and Clinicopathological Features in OED and OSCC

There was no correlation between METTL3 expression and gender, age, the site of the lesions, the T-stage of OSCC, the histology of OED (low- and high-grade OED), and histology of OSCC (well-differentiated and moderately-to-poorly differentiated OSCC) ( *p* > 0.05) ( Table 4 ).

**Table 4:** Correlations of the METTL3 expression and clinicopathologic data in OED and OSCC

Clinicopathological features	OED		*p* -Value	OSCC		*p* -Value
	Low METTL3 expression *n* (%)	High METTL3 expression *n* (%)		Low METTL3 expression *n* (%)	High METTL3 expression *n* (%)	
Age (years old)						
≤ 60	1 (5)	14 (70)	0.14	1 (5)	9 (45)	1.0
> 60	2 (10)	3 (15)		0 (0)	10 (50)	
Gender						
Male	1 (5)	7 (35)	1.0	1 (5)	16 (80)	1.0
Female	2 (10)	10 (50)		0 (0)	3 (15)	
Site of lesions						
Tongue	1 (5)	9 (45)	1.0	0 (0)	5 (25)	1.0
Others a	2 (10)	8 (40)		1 (5)	14 (70)	
T stage of OSCC						
T1	N/A	N/A	N/A	1 (5)	11 (55)	1.0
T2 and T3	N/A	N/A		0 (0)	8 (40)	
Histology of OED						
Low-grade OED	3 (15)	7 (35)	0.21	N/A	N/A	N/A
High-grade OED	0 (0)	10 (50)		N/A	N/A	
Histology of OSCC						
Well differentiated	N/A	N/A	N/A	1 (5)	15 (75)	1.0
Moderately-to-poorly differentiated	N/A	N/A		0 (0)	4 (20)	

Abbreviations: H-score, histochemical score; METTL3, methyltransferase-like 3; N/A, not available; OED, oral epithelial dysplasia; OSCC, oral squamous cell carcinoma.

*p* < 0.05 indicated statistically significant differences.

Note: Low METTL3 expression was defined as H-score ≤ 150 and high METTL3 expression was defined as H-score > 150.

a Others included gingiva (2 cases), buccal mucosa (4 cases), and hard palate (4 cases) for OED and gingiva (11 cases), buccal mucosa (2 cases), hard palate (1 cases), and floor of the mouth (1 cases) for OSCC.

## Discussion

Recently, METTL3 has been reported to be involved in tumor cell proliferation, invasion, and migration of various human cancers, including OSCCs. 9 21 However, there is no information regarding the possible role of METTL3 in OED. In the present study, we attempted to evaluate the METTL3 immunohistochemical expression of NOM, OED, and OSCC. We also analyzed the METTL3 expression between low- and high-grade OED, and between well-differentiated and moderately-to-poorly differentiated OSCC. The gradual increase in percentage of METTL3 positive cells and H-score was observed from the NOM to low- and high-grade OED to OSCC samples. The METTL3 staining intensity was also increased in OED and OSCC compared with NOM samples. These results would suggest the possible roles of METTL3 in OSCC tumorigenesis.

In the present study, the observation of the nuclear localization of METTL3 in OED and OSCC cases was generally consistent with numerous tumors, including colorectal cancer, 11 gastric adenocarcinoma, 22 esophageal and skin SCC. 23 24 Zhou et al and Wu et al reported that METTL3 was found to be highly expressed and colocalized with the proliferation marker K _i_ -67 and β-catenin in esophageal SCC and OSCC, respectively. These results suggested that METTL3 may be associated with the proliferation of dysplastic cells in esophageal SCC and OSCC. 17 24 In agreement with previous studies, 14 15 16 17 18 it was found that METTL3 was overexpressed in OSCC when compared with NOM. We also observed that the expression of METTL3 in NOM located the basal, parabasal, and lower layers of the epithelium. Nevertheless, their expression was increased in the spinous layer and upper layers of dysplastic epithelium of OED and in both peripheral and central cells of the tumor islands of OSCC. The difference in the expression pattern of METTL3 among the groups may further support METTL3 involvement in the pathogenesis of abnormal epithelial proliferation in OSCC and OED.

Previous studies have been proposed on the molecular mechanism of the role of METTL3 in OSCC. METTL3 promoted OSCC tumorigenesis by enhancing m6A modification of c-Myc mRNA and maintained its stability, thus leading to the proliferation, invasion, and migration of OSCC cells. 15 The upregulation of METTL3 was found to activate p38 in OSCC, resulting in the OSCC cell proliferation and growth. 16 Liu et al reported that METTL3 inducing m6A modification of a cancer stem cell transcript BMI1 mRNA. 14 METTL3 promoted the translation of BMI1 under the cooperation with m6A reader IGF2BP1, leading to proliferation, invasion, and migration of OSCC cells. 14 METTL3 also promotes OSCC proliferation, invasion, and migration and attenuated the activation CD8+ T cells by enhancing m6A modification and expression of PRMT5 and PD-L1. 18 In our study, we found that METTL3 may promote tumor progression as we observe an increase in METTL3 expression from NOM, low- and high-grade OED, and OSCC, respectively. Because we only aimed to investigate the expression of METTL3 among NOM, OED, and OSCC, we did not investigate the possible target genes of METTL3 in OED in this study. Further research is necessary to identify the target genes of METTL3 in OED to help understand the role of METTL3 in OED progression to OSCC.

Increased METTL3 expression was associated with the advanced T stage, lymph nodes metastasis, and poor survival rates in OSCC. 14 15 16 17 However, we did not find any correlations between METTL3 expression and on the clinical data of the study population. This may be due to limited sample size of this study. Additionally, some clinical data on OED and OSCC are based on chart reviews that may be missing and cannot be analyzed, such as TNM staging of OSCC and risk factors for precancerous lesions and OSCC. Therefore, the study of METTL3 expression with the larger sample size is required for clarification and definite results. However, to the best of our knowledge, this is the first study to evaluate the expression of METTL3 in the precancerous lesions of the epithelial tumors. A gradual increase in METTL3 expression together with an increase in the severity of histopathological grading was observed in this study, suggesting the possible roles of METTL3 in oral malignant transformation. Thus, the preliminary results from this study may help provide a better understanding on the possible role of METTL3 in OED and OSCC.

## Conclusion

This study showed that METTL3 expression in OED and OSCC was more outstanding than NOM, suggesting possible roles for OED and OSCC pathogenesis. Additionally, an increase in nuclear METTL3 expression from NOM to low- and high-grade OED to OSCC specimens were observed. The results implied that METTL3 may be a potential biomarker for detecting malignant transformation of OED. However, future studies are still needed to clarify the molecular mechanism of the role of METTL3 in oral carcinogenesis.
